# Liposome drug delivery strategies: state-of-the-art for combating bacterial biofilms

**DOI:** 10.3389/fmicb.2026.1899406

**Published:** 2026-07-30

**Authors:** Tao Lin, Haifeng Liu, Wei He, Wei Hu, Huifang Jiang, Zhiyang Xu, Jintao Wei, Mingdong Yang, Haibin Dai, Junjun Xu

**Affiliations:** 1 Department of Ultrasound in Medicine, The Second Affiliated Hospital, Zhejiang University School of Medicine, Hangzhou, China; 2 Department of Pharmacy, The Second Affiliated Hospital, Zhejiang University School of Medicine, Hangzhou, China; 3 Department of Infectious Diseases, The Second Affiliated Hospital, Zhejiang University School of Medicine, Hangzhou, China; 4 Department of Emergency Medicine, The Second Affiliated Hospital, Zhejiang University School of Medicine, Hangzhou, China

**Keywords:** anti-infection therapy, bacterial bioflims, bioflim barrier, drug delivery systems, liposome

## Abstract

Bacterial biofilms pose a significant challenge in clinical settings due to their inherent resistance to conventional antibiotics and host immune responses. This review comprehensively discusses the application of liposomal drug delivery systems as a cutting-edge strategy to combat biofilm-associated infections. It begins by outlining the biological characteristics of biofilms, including their formation process and the protective extracellular polymeric substance (EPS) matrix. The manuscript then focuses on the advantages of liposomes, such as their nanoscale size effect, modifiable surface properties, and sustained-release capabilities, which enhance penetration through the EPS and improve drug accumulation at infection sites. Furthermore, it details species-specific strategies against pathogens like *Staphylococcus aureus*, *Pseudomonas aeruginosa*, and *Escherichia coli*. The review also covers advanced mechanisms including quorum sensing interference, stimuli-responsive release (pH, enzyme, temperature), and synergistic therapies combining antibiotics with antimicrobial peptides or natural products. Finally, it summarizes current clinical studies and outlines future prospects for precision targeting and multi-stimuli responsive systems to improve the treatment of chronic biofilm infections.

## Highlights

1. The growth of bacterial biofilms is divided into five distinct stages. Tissues infected by biofilms possess a unique microenvironment characterized by altered pH, redox gradients, hypoxia, and characteristic bacterial toxins. As a mature nanoscale drug delivery system, liposomes offer substantial advantages against biofilm-associated infections through their size effects, controllable surface charge and hydrophilicity/hydrophobicity, sustainedrelease characteristics, capacity for functional modification, and high biocompatibility.
2. This review comprehensively summarizes the latest targeted controlled-release strategies for bacterial biofilms, including photodynamic/photothermal therapy (PDT/PTT) combinations, pH-responsive, thermosensitive, and ultrasound-triggered systems. It further introduces combined modification strategies utilizing exosome membranes and engineered biofilm membranes, as well as quorum sensing interference approaches, and delineates the synergistic logic of enzyme-assisted penetration coupled with intelligent drug release.
3. This review innovatively presents pathogen-specific differentiated strategies:
  - For *Staphylococcus aureus*/MRSA, it covers protein corona-mediated targeting, antimicrobial peptide mimetics, DNA aptamers, and cationic charge attraction.
  - For *Pseudomonas aeruginosa*, it discusses glycomimetic modification competing with bacterial lectins, efflux pump inhibitor combinations, and ultrasoundselectively targeted liposomes.
  - For *Escherichia coli*, it addresses charge modulation based on surface ionization properties and outer membrane vesicle hybridization.
  - For other pathogens including *Mycobacterium avium*, *Acinetobacter baumannii*, *Klebsiella pneumoniae*, *Helicobacter pylori*, and oral bacterial communities, it proposes innovative strategies such as four-enzyme cocktail therapy, antimicrobial peptide 2K4L, EDTA chelation, lignin mucoadhesive systems, and pyrophosphate-hydroxyapatite anchoring.
4. This review systematically catalogs clinically approved and investigational formulations, including amikacin liposome inhalation suspension, amphotericin B liposomes, and polymyxin liposomes. It further summarizes multiple antibiotic coloading strategies, encompassing antibiotic-antimicrobial peptide, antibiotic-phage, antibiotic-natural product, and photothermal combination therapies, clearly identifying the future research direction as precision targeting and efficient biofilm disruption.

## 1 Introduction to bacterial biofilms

### 1.1 Overview of bacterial biofilms

Biofilms are microbial communities formed by bacteria sensing external environmental stimuli and colonizing solid surfaces through unique adhesion mechanisms (Carter et al., 2019). These microorganisms aggregate via flagella, pili, and other appendages. Upon aggregation, they abundantly secrete extracellular polymeric substances (EPS), including exopolysaccharides, proteins, lipids, and extracellular DNA. The cross-linking of EPS forms a highly organized, tightly structured membranous three-dimensional complex that encapsulates bacteria within monolayer or multilayer biofilm architectures (Sauer et al., 2022). Such biofilm structures promote bacterial community adhesion and proliferation, enhance the sharing of nutrients and genetic material, generate physicochemical barrier effects, and produce unique virulence factors. Meanwhile, bacteria within biofilms continuously proliferate and disperse from the biofilm to invade the host, causing persistent infections that further exacerbate the difficulty of treating chronic bacterial infectious diseases, making such conditions particularly refractory to eradication (Ragupathi et al., 2024).

### 1.2 Biofilm formation process

**Figure 1:**
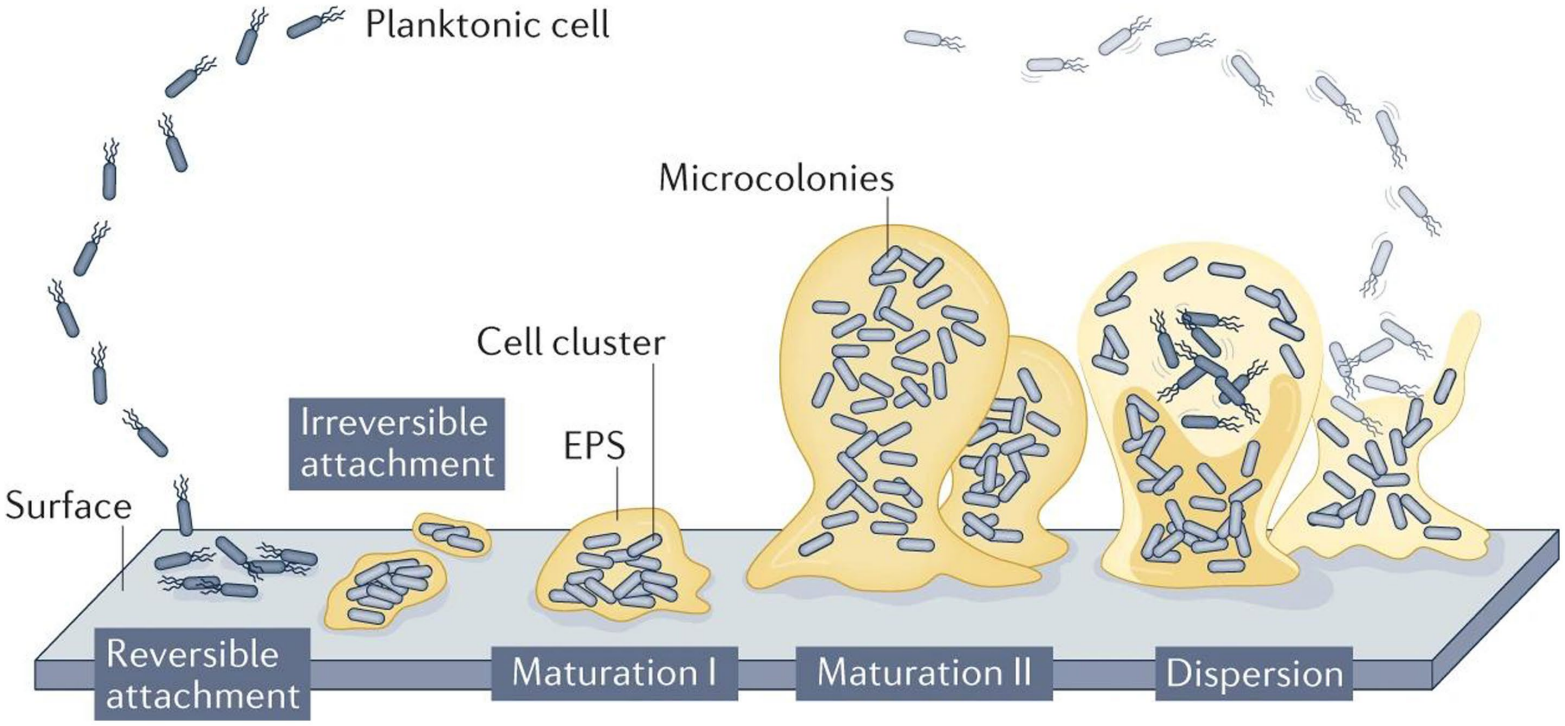
Schematic illustration of biofilm formation mechanisms (Sauer et al., 2022).

Biofilms achieve sustained proliferation and dissemination through a dynamic cyclic process encompassing five distinct stages: surface adhesion (subdivided into reversible and irreversible adhesion), microcolony formation (first maturation stage), full maturation (second maturation stage), and ultimate dispersal (Ma et al., 2022) (Figure 1).

The surface adhesion stage represents the first critical phase, characterized by the transition of bacteria from planktonic to adherent states. This stage can be further divided into reversible and irreversible adhesion (Sauer et al., 2022). During reversible adhesion, bacteria employ various extracellular organelles and proteins to sense and attach to solid surfaces. Interfacial interactions at this stage are primarily governed by electrostatic forces and van der Waals forces. These relatively weak interactions enable bacteria at this stage to either proceed to subsequent biofilm formation stages or revert to their previous planktonic state when necessary (Renner and Weibel, 2011). As the number of adherent bacteria increases, adhesin molecules are secreted in substantial quantities, facilitating stronger attachment between bacteria and the surface, thereby transitioning adherent bacteria into the irreversible adhesion stage (Berne and Brun, 2019). Biofilm formation can be interfered with at this stage by preventing the transition from planktonic to adherent states. Commonly employed intervention strategies include: (1) competitive inhibition of adhesion: glycomimetic liposomes compete for binding to bacterial surface lectins (LecA/LecB), thereby blocking initial bacterial adhesion to host cells; (2) electrostatic interference: charged liposomes bind to bacterial surfaces through electrostatic interactions, preventing their contact with colonization surfaces; (3) surface modification; (4) vaccination for prevention; and (5) quorum sensing interference: blocking AHL signaling molecules to prevent bacteria from sensing environmental cues and initiating adhesion gene expression.

Following surface adhesion, bacterial cells begin to proliferate and gradually form bacterial aggregates, marking the onset of the first maturation stage. Through intercellular interactions and signal transduction (termed quorum sensing), bacteria can perceive environmental changes and modulate their metabolic behaviors and gene expression. Concurrently, aggregates continuously release additional signaling molecules to recruit planktonic bacteria for aggregation and adhesion. As bacterial aggregation and proliferation proceed, bacteria within the aggregates commence synthesizing and secreting extracellular polymeric substances (EPS) (Rather et al., 2021; Liu et al., 2017; Nair et al., 2018). Microcolonies at this first maturation stage can be targeted by inhibiting continued EPS synthesis and degrading early-stage matrices. Commonly employed methods include direct delivery of enzymes or plant extracts to block and decompose EPS, phage conjugation-mediated EPS degradation via depolymerases, and quorum sensing interference, among others.

Progressively developing microcolonies continuously secrete EPS and grow vertically, forming complex three-dimensional structures indicative of the biofilm’s entry into the second maturation stage. At this stage, biofilms typically exhibit a three-layer architecture comprising an inner regulatory layer, a middle microbial basal layer, and an outer layer (Bai et al., 2023). Bacterial cell volumes and metabolic activities vary across these regions:

outer-layer bacteria possess abundant nutrient supply and high metabolic activity, whereas bacteria in the inner regulatory layer are nutrient-deprived, predominantly dormant, exhibit low metabolic activity, and reduced drug sensitivity. This represents the principal stage of bacterial biofilm existence and the primary focus of targeted research. The main intervention objectives are to penetrate the dense EPS barrier, target deepseated dormant bacteria, and enhance local drug concentrations. Commonly employed strategies include: (1) physical penetration: ultrasound activation, photodynamic/photothermal therapy (PDT/PTT); (2) intelligent microenvironmentresponsive drug release: pH-, enzyme-, redox-, thermal-, and ultrasound-responsive modalities; (3) penetration-enhancing targeting strategies: particle size optimization, charge modulation, and active targeting (e.g., protein corona-mediated, DNA aptamer, OMV hybridization); (4) synergistic killing: combination killing regimens involving antibiotics with antimicrobial peptides, phages, natural products, adjuvants, and enzymes; and (5) metabolic and immunomodulation: immune activation, macrophage polarization, among others.

**Figure 2:**
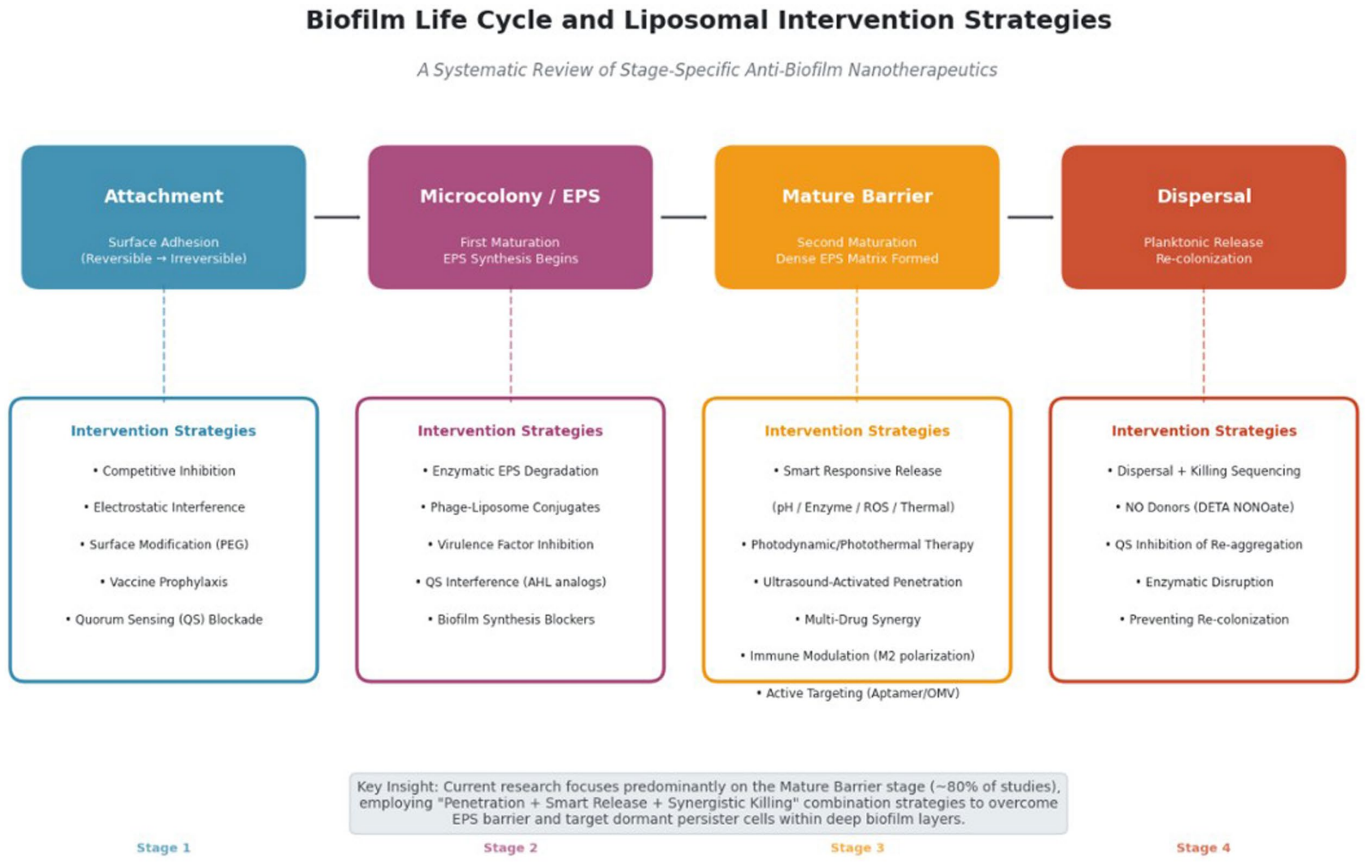
Full-cycle intervention strategies and commonly used methods.

As bacterial numbers and aggregate volumes increase, the biofilm ultimately enters the dispersal stage, characterized by the outermost bacteria reverting to planktonic states accompanied by EPS disintegration. Dispersed bacteria subsequently colonize new sites and establish novel biofilm structures, thereby reinitiating the cycle (Santiago et al., 2015). For bacteria in the dispersal stage, the primary objective is immediate bactericidal action to prevent disseminated infections and recolonization. Principal methods include quorum sensing inhibition and sequential drug release, among others. Whole-cycle intervention strategies and commonly employed methods are presented in the Figure 2.

### 1.3 Characteristics of bacterial biofilms

As described above, addressing biofilm-associated infections requires overcoming the following key challenges:

- Immune Evasion. The abundant extracellular polymeric substance (EPS) serves as an immunological barrier, isolating bacteria from the host immune system and preventing effective infiltration of immune cells. Furthermore, bacteria can secrete biological factors that directly attack host immune cells, thereby resisting immune responses (Scherr et al., 2015).
- Physicochemical Barrier. The physicochemical barrier formed by biofilms impedes the penetration of antibiotics and other chemical agents, and possesses certain neutralizing capabilities. Moreover, biofilms exhibit remarkable resistance to changes in external physicochemical conditions, including osmotic pressure gradients and shear stress (Ricciardi et al., 2018). The dense EPS matrix can trap or neutralize bactericides before they reach the inner bacterial layers, and the structural heterogeneity of biofilms renders physical removal (such as debridement and irrigation) often incomplete, leading to rapid regeneration (Stewart and Costerton, 2001).
- Antibiotic Resistance or Reduced Susceptibility. On one hand, bacteria in deep regions primarily exist in a dormant state characterized by low metabolic activity and nutrient deprivation, resulting in reduced drug susceptibility. On the other hand, bacteria within biofilms exhibit upregulated expression of antibiotic resistance genes and are more prone to acquiring such resistance genes. This severely limits the efficacy of conventional monotherapy with antibiotics and directly exacerbates the global antimicrobial resistance crisis (Wang et al., 2026; Penesyan et al., 2015).
- Recurrent Infection. The persister state of bacteria within biofilms leads to direct infection and the secretion of characteristic toxins. Even after apparent clinical remission, residual biofilm-embedded persister cells can reinitiate the infection cycle, necessitating prolonged or repeated antibiotic courses, and often requiring invasive removal of infected implants or tissues (Ragupathi et al., 2024).

Current research has revealed that tissues infected by biofilms possess a unique microenvironment distinctly different from normal tissues, mainly manifested in the following aspects:

1. Low pH: Due to immune responses and anaerobic glycolysis, the pH of the bacterial biofilm microenvironment typically ranges between 5 and 7;
2. Redox environment: Another characteristic of biofilms is the simultaneous presence of high concentrations of glutathione and reactive oxygen species (ROS) at the infection site, where glutathione concentration is approximately 0.1 to 10 mM, and hydrogen peroxide (H₂O₂) is typically around 100 μM;
3. Bacterial toxins: Bacteria secrete characteristic toxins, such as pore-forming toxins, pyocyanin, and enterotoxins;
4. Nutrient deprivation and hypoxia: Due to restricted substance exchange caused by biofilm formation, nutrients and oxygen in deep biofilm regions become increasingly depleted (Huang et al., 2022).
5. Elevated reactive oxygen species levels: The biofilm microenvironment is characterized by elevated levels of ROS and H₂O₂, which can promote biofilm formation by enhancing EPS production (such as alginate in *Pseudomonas aeruginosa*) and induce oxidative stress to damage host tissues (Xiu et al., 2022).
6. High lactate: Anaerobic glycolysis and bacterial fermentation within biofilms produce substantial amounts of lactate, further acidifying the local microenvironment and creating a lactate gradient that selects for acid-tolerant species and reinforces biofilm persistence.
7. Ion gradients (Ca^2+^/Mg^2+^): Biofilms accumulate divalent cations such as Ca^2+^ and Mg^2+^, which stabilize the EPS matrix by cross-linking with polysaccharides and extracellular DNA (eDNA). Such ion gradients contribute to maintaining the structural integrity of biofilms (Allison, 2003).

## 2 Selection of anti-biofilm drug delivery systems

Currently, antibiotic therapy remains the primary approach for clinical treatment of bacterial infections worldwide. However, the use of antibiotics faces a series of clinical challenges, most notably the increasingly severe problem of bacterial resistance. Based on the growth characteristics and biological properties of bacterial biofilms described above, it is evident that biofilms significantly exacerbate antibiotic resistance. The underlying reasons are as follows: (1) Most antibiotics must enter bacterial cells to exert their effects, yet biofilms impede this process; (2) Enhanced intercellular communication within bacterial biofilms promotes the dissemination of resistance genes (Feng et al., 2022; Andersson et al., 2021; Privalsky et al., 2021). Therefore, a nanotechnology strategy capable of simultaneously delivering antibiotics into bacterial cells represents an ideal solution to address the clinical challenges of antibiotic therapy.

Currently, mainstream nanostructured drug delivery systems (NDDS) can be classified by composition into lipid-based nanoparticles, polymer-based nanoparticles, protein nanoparticles, carbon-based nanoparticles, metal-based nanoparticles, and biocomposite nanoparticles (Kumar et al., 2026). Based on structural morphology, they can be further categorized into liposomes, micelles, metal–organic frameworks, nanocages, and exosomes. Each of these NDDS possesses unique advantages and limitations. For example, liposomes readily penetrate biofilms but are prone to drug leakage; micelles exhibit excellent self-assembly capabilities but are susceptible to metabolic clearance; inorganic nanoparticles have stable structures with superior optical or magnetic properties but are difficult to eliminate from the body; exosomes demonstrate good biocompatibility and homing capabilities but are challenging to isolate (Akomolafe and Akinsiku, 2025; Kantesaria and Panda, 2026). Currently, researchers worldwide are employing novel NDDS or combinations of multiple NDDS to achieve more stable and efficient drug delivery. In particular, liposomes have attracted considerable attention due to their ease of functionalization through modification of lipid composition, including the incorporation of fusogenic lipids to confer membrane fusion capabilities. This ability to facilitate transmembrane delivery of encapsulated cargo has been widely applied in eukaryotic cell transfection and has shown tremendous potential in addressing the inherent antimicrobial resistance of Gram-negative bacteria (Scheeder et al., 2023).

Compared to free antibiotics, the advantages of liposomal anti-biofilm strategies include:

(1) Nanoscale size effect (50–200 nm) enhances the ability to penetrate the EPS matrix; (2) Modifiable surface charge and hydrophobicity/hydrophilicity optimize interactions with bacteria; (3) Sustained-release characteristics maintain therapeutically effective local drug concentrations; (4) Functionalization enables active targeting and stimuli-responsive release; (5) Reduced systemic toxicity and side effects (Gandhi and Shastri, 2024). Gkartziou et al. (2024) evaluated the antimicrobial activity of neutral and negatively charged liposomes loaded with daptomycin against planktonic bacteria and biofilms. Measurements of biofilm biomass (crystal violet assay), biofilm viability (MTT assay), and growth curves demonstrated that liposomal encapsulation significantly enhanced the preventive and eradication efficacy of daptomycin against staphylococcal biofilms.

This review primarily focuses on the design of liposomes as the principal NDDS, with particular emphasis on their targeting, diagnostic, controlled-release, and other unique delivery functionalities. The latest research progress is systematically reviewed from three dimensions: mechanism of action, species-specific strategies, and innovative design.

## 3 Applications of liposomes in bacterial biofilms

### 3.1 Species-specific strategies

#### 3.1.1 *Staphylococcus aureus/methicillin-resistant Staphylococcus aureus* (*S. aureus*/MRSA)

For *Staphylococcus aureus*, the application of liposomes as anti-biofilm drug delivery carriers has been extensively investigated. Natsaridis et al. (2025) systematically examined the effects of cholesterol content, liposome concentration, and PEG surface modification on the anti-staphylococcal activity of moxifloxacin-loaded liposomes, aiming to optimize liposomal formulations to enhance antimicrobial efficacy. Furthermore, the use of liposomes alone or in combination with other carriers has attracted considerable research interest in recent years.

The surface charge of liposomes profoundly influences their binding to specific bacterial components, thereby facilitating the internalization of encapsulated cargo, particularly their traversal through bacterial biofilms into the intracellular environment. Notably, this process has been demonstrated to be bacterial species-specific in the work of Scheeder et al. (2023). Ion charge-mediated targeting strategies are frequently employed against MRSA. Guo et al. (2021) designed curcumin-loaded cationic liposomes (C-LS/Cur) that bind to negatively charged *S. aureus* through electrostatic interactions, thereby enhancing curcumin accumulation in drug-resistant *S. aureus*. Pagano et al. (2022) developed liposomes containing a cationic cholesterol derivative (DC-Chol) and glycosylated lipid (GL4) to investigate the roles of cationic charge and glycosylation in liposome-bacterial cell interactions, and utilized these formulations to encapsulate resveratrol for the treatment of *S. aureus* infections.

The unique protein corona constitutes another important therapeutic target. Shao et al. (2022) demonstrated that the protein corona can modulate liposome-bacteria interactions. Specifically, anionic liposomes containing DSPG exhibited enhanced binding affinity to.

**Figure 3:**
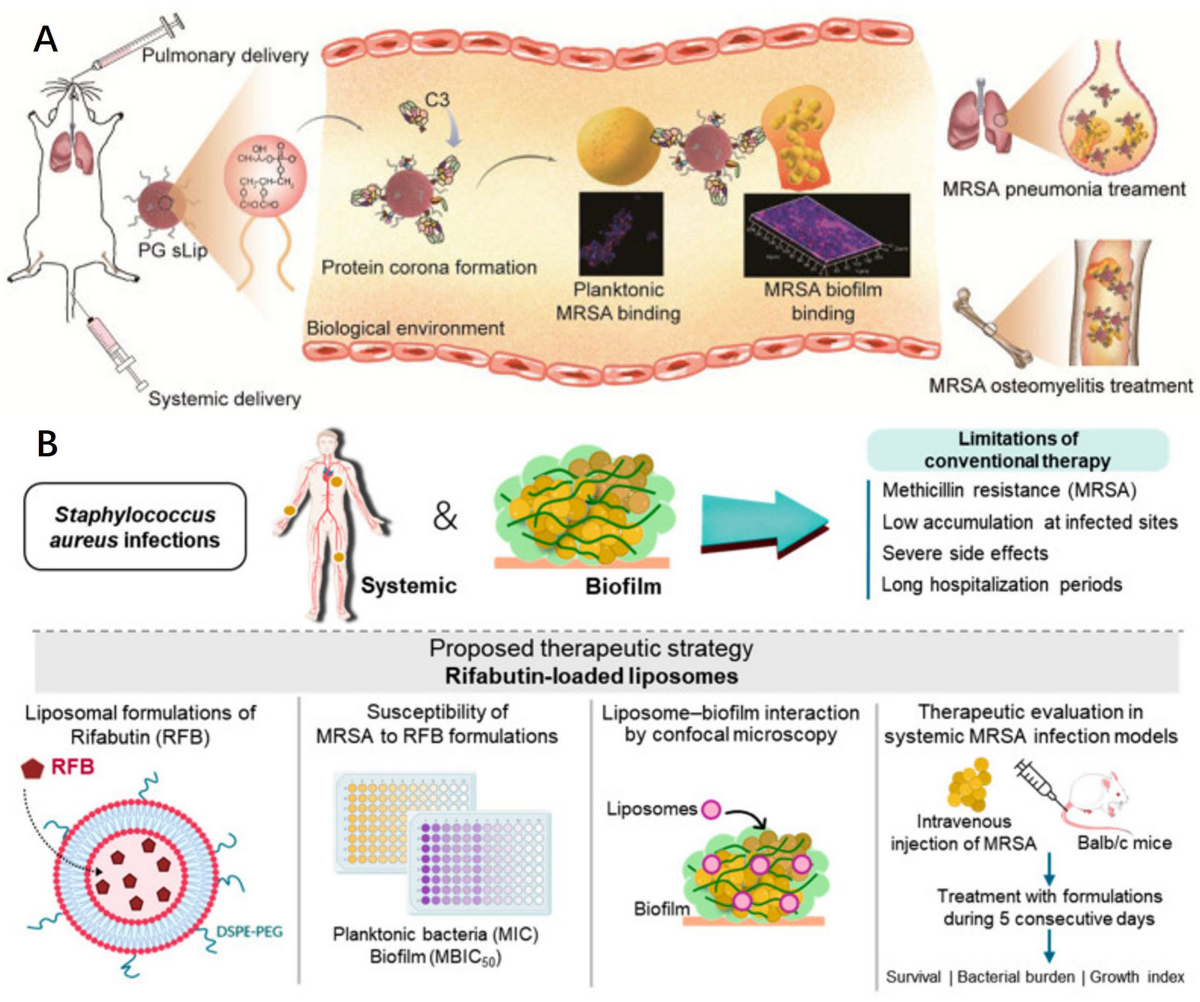
**(A)** Schematic illustration of protein corona-mediated modulation of liposome-bacteria interactions. **(B)** Schematic illustration of rifabutin liposomal formulations targeting MRSA-C1 strain and comparison with vancomycin (Shao et al., 2022; Pinho et al., 2024).

MRSA through complement deposition from serum, and showed therapeutic effects in MRSA osteomyelitis and pneumonia mouse models. This study revealed that the protein corona is not merely a barrier but can also serve as a mediator for active targeting (Figure 3A).

Antimicrobial peptides have also been utilized as targeting and therapeutic agents. Hemmingsen et al. (2023) developed liposomes loaded with 7e-SMAMP (small molecule antimicrobial peptide mimetic), which completely eradicated *S. aureus* biofilms at concentrations above 6.25 μg/mL. This system simultaneously reduced inflammatory responses in mouse macrophages by approximately 30%, demonstrating considerable therapeutic potential in the treatment of chronic wound infections. Compared to natural antimicrobial peptides, SMAMP mimetics exhibit superior stability and lower immunogenicity.

Targeting the unique acidic microenvironment of MRSA, Zang et al. (2024) prepared a baicalein-loaded NDDS modified with polyhexamethylene guanidine (PHMG) and hyaluronic acid (HA). The researchers applied this system to the treatment of MRSAinduced subcutaneous abscesses on the dorsal back and thigh muscle infection models in mice. The presence of guanidinium groups in HA/P/BAI-lip conferred satisfactory bacterial targeting capability and favorable pH sensitivity to the liposomes. Bacterial lipase promoted the hydrolysis of soybean phosphatidylcholine (SPC) in the liposomes. HA modification on HA/P/BAI-lip guided the drug system precisely to infection sites where CD44 is overexpressed due to inflammation. The characteristic low-pH microenvironment at infection sites induced liposome swelling and subsequent degradation.

Other researchers have adopted alternative strategies. For example, Ommen et al. (2022) developed DNA aptamer-targeted liposomal NDDS, screening DNA aptamers that bind to *S. aureus* cells to achieve localized accumulation and delivery of low-dose antibiotics within biofilms. Additionally, some researchers have utilized natural products as unique targeting and antimicrobial agents. Pinho et al. (2024) developed rifabutin liposomal formulations that exhibited favorable antimicrobial activity against the MRSA-C1 strain compared to vancomycin (Figure 3B). Wen et al. (2024) developed moringin-loaded chitosancoated liposomes (MR-CS-LPs) that demonstrated potent antimicrobial activity against *S. aureus*, providing a novel platform for the application of natural products in antimicrobial therapy. Mohamed et al. (2025) developed luteolin-loaded PEGylated cerosomes targeting MRSA-induced skin and soft tissue infections. Given the increasingly severe threat posed by the spread of antimicrobial resistance, cerosomes, as a novel lipid-based nanocarrier, offer enhanced skin penetration and retention capabilities.

#### 3.1.2 *Pseudomonas aeruginosa* (*P. aeruginosa*)

*Pseudomonas aeruginosa*, formerly known as Bacillus pyocyaneus, is the representative species of the Pseudomonas genus, a common Gram-negative opportunistic pathogenic rod. A direct therapeutic approach involves co-encapsulation of antibiotics; Milani et al. (2023) developed imipenem/cilastatin nanoliposomes, and evaluation demonstrated that this liposomal formulation enhanced anti-*P. aeruginosa* efficacy.

**Figure 4:**
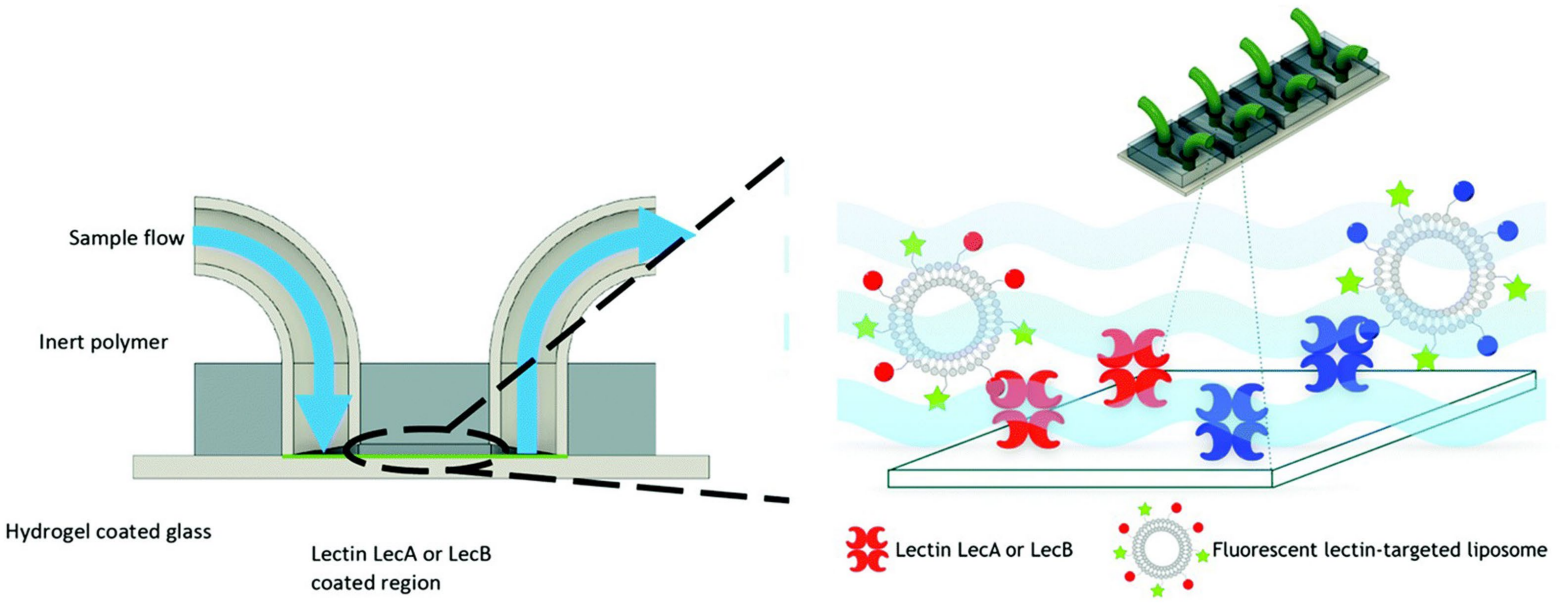
Mechanism diagram of sugar-mimetic liposomes targeting *Pseudomonas aeruginosa* extracellular lectin competitive binding to inhibit biofilm formation by lectin (Metelkina et al., 2022).

Glycomimetic modification represents another major strategy. Metelkina et al. (2022) developed glycomimetic liposomes targeting *P. aeruginosa* extracellular lectins LecA and LecB. Lectins play crucial roles in biofilm formation and maintenance; glycomimetic liposomes inhibit biofilm formation through competitive lectin binding, thereby providing a novel therapeutic strategy for *P. aeruginosa* infections (Figure 4). Alluhaim et al. (2024) utilized Manuka honey as a surfactant to prepare liposomes encapsulating methylglyoxal and tobramycin. Honey itself possesses antimicrobial and antiinflammatory activity, exerting synergistic effects with antibiotics; this formulation achieved sustained drug release over 24 h with tobramycin retention exceeding 99%.

Natural product modification has also been frequently adopted. Sadeghi Mohammadi et al. (2022) developed gentamicin and curcumin co-loaded lipid-polymer hybrid nanoparticles to synergistically enhance anti-biofilm efficacy, addressing chronic pulmonary infections and disease recurrence caused by *Pseudomonas aeruginosa* residing intracellularly and within the biofilm matrix in cystic fibrosis patients. Hemmati et al. (2024) developed stearylamine (SA)-modified quercetin (QCT)-loaded niosomes as an NDDS, enhancing the antimicrobial and anti-biofilm activity of QCT against both standard and clinical strains of *P. aeruginosa*. Cationic surface modification improved interactions with bacterial membranes.

Thorn et al. (2021) developed a biomimetic nano-liposomal tobramycin liquid crystalline nanoparticle (LCNP) formulation that effectively eradicated cystic fibrosis-related *P. aeruginosa* biofilms, addressing the severely diminished efficacy of tobramycin in biofilm infections due to the limited permeability of the biofilm matrix.

**Figure 5:**
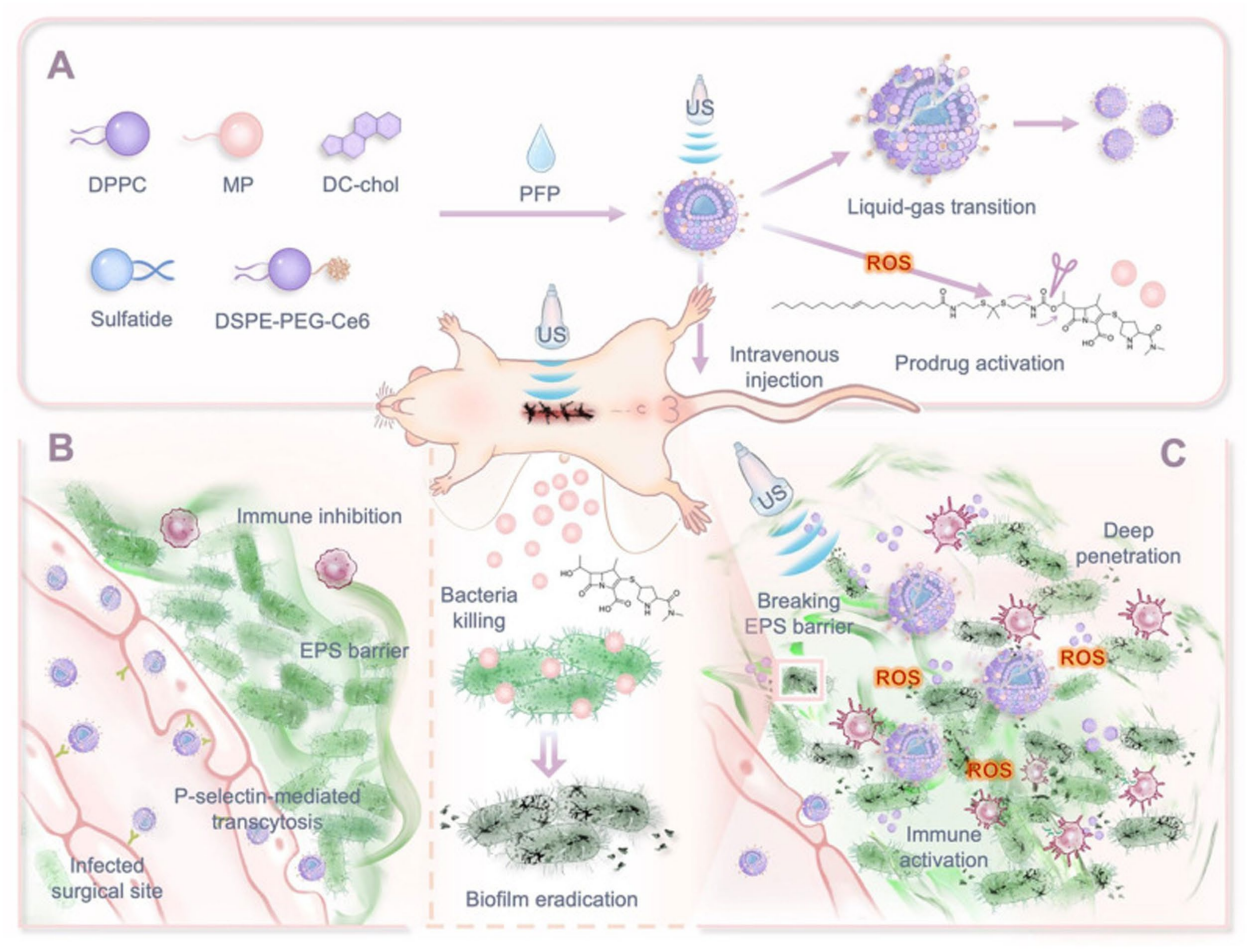
Schematic representation of SPCMPL for efficient biofilm eradication in the deep BSSI model. **(A)** The fabrication of SPCMPL, alongside its size transformation and prodrug activation upon ultrasonic irradiation. **(B)** SPCMPL actively targeted the highly expressed P-selectin in vascular endothelial cells at BSSI site and utilized P-selectin-mediated transcytosis to enrich drug accumulation and reach the infection site. **(C)** Ultrasonic cavitation broke the EPS barrier to promote SPCMPL deep penetration, which worked with immune activation to destroy bacteria and clear biofilm-associated infections (Liu et al., 2025).

Liu et al. (2025) addressed the challenge posed by the dense EPS barrier of *P. aeruginosa* that impedes drug enrichment and penetration, developing sonodynamic responseactivated, P-selectin-precisely targeted liposome-loaded drugs, achieving deep penetration within EPS and generating immune activation mechanisms (Figure 5).

Gbian and Omri (2021) designed a liposomal formulation co-loading gentamicin and erythromycin with the broad-spectrum efflux pump inhibitor phenylalanine-arginine βnaphthylamide (PABN). Results demonstrated that compared to monotherapy, this liposomal formulation showed superior therapeutic outcomes in pulmonary infections of cystic fibrosis patients.

Furthermore, phage research targeting *P. aeruginosa* is relatively mature, and scholars worldwide are extensively investigating phage-liposome combination strategies.

#### 3.1.3 *Escherichia coli* (*E. coli*)

*E. coli* biofilms are commonly found in urinary tract and gastrointestinal infections.

The outer membrane of *E. coli* carries a negative charge; therefore, cationic liposomes can enhance binding through electrostatic attraction. However, cytotoxicity must be carefully considered. Many antibiotics are natural antimicrobial products of bacteria, many of which are inherently positively charged and poorly soluble. In this regard, Viera Herrera et al. (2024) encapsulated the bacterially produced dipeptide antimicrobial agent Thuricin CD in anionic liposomes, utilizing the binding capacity and stability of anionic liposomes to achieve precise gastrointestinal delivery. This method protected Thuricin CD from enzymatic degradation in the gastrointestinal tract, while the inherent positive charge of Thuricin CD enabled it to target *E. coli* upon release. Hybridization with outer membrane vesicles (OMVs)—fusing bacterial OMVs with liposomes—has been demonstrated to enhance interactions with homologous bacteria (Privalsky et al., 2021).

#### 3.1.4 Other species and mixed biofilms

**Figure 6:**
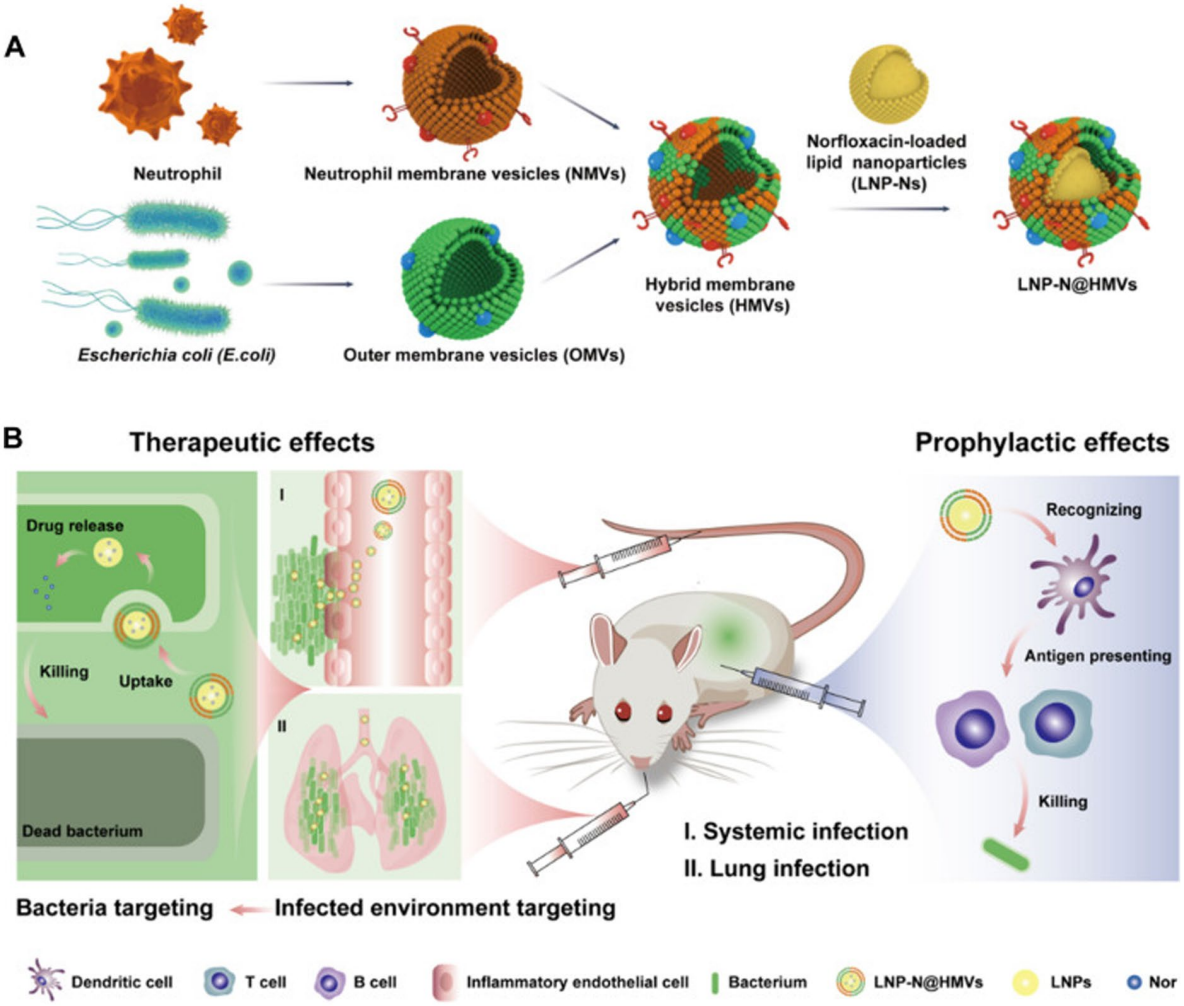
Schematic illustrations of the construction and application of LNP-N@HMVs. **(A)** Construction of hybrid cell membrane–coated antibiotic delivery system LNP-N@HMVs. **(B)** Application of LNP-N@HMVs in dual-targeted treatment and prophylaxis of bacterial infections (Peng et al., 2024).

Targeting Gram-negative bacteria, Peng et al. (2024) successfully prepared neutrophilbacteria lipid nanoparticle@hybrid membrane vesicles (LNP@HMV) for dual-targeted antibiotic delivery, simultaneously activating humoral and cellular immunity to prevent Gram-negative bacterial infections. LNP@HMV was obtained by coating lipid nanoparticles (LNPs) with a hybrid membrane composed of bacterial outer membrane vesicles (OMVs) and neutrophil membrane vesicles (NMVs), capable of simultaneously targeting inflammatory vascular cells and Gram-negative bacteria, and exerting antibacterial vaccine effects through activation of specific humoral and cellular immunity to prevent bacterial infections (Figure 6).

Targeting mycobacteria, Bartlett et al. (2024) developed a four-enzyme cocktail capable of catalytically attacking the triple-layered envelope of mycobacteria. This cocktail was delivered to macrophages through the targeted liposomal system ENTX_001. Endolytix Cocktail 1 (EC1) contains lytic enzymes LysA and LysB derived from mycobacteriophages, as well as *α*-amylase and isoamylase, which can degrade the mycobacterial envelope extracellularly. LysA protein family members derived from mycobacteriophages have been demonstrated to cleave the peptidoglycan layer, while LysB is an esterase that hydrolyzes the ester bond between arabinogalactan and mycolic acid. The addition of amylases capable of degrading the extracellular capsule of *Mycobacterium tuberculosis* addressed the substrate accessibility challenge for exogenous LysA and LysB. This enzyme cocktail exhibited bactericidal activity against both rapidly and slowly growing non-tuberculous mycobacteria (NTM) and.

*Mycobacterium tuberculosis* strains *in vitro*. Nitric oxide (NO) is a natural antimicrobial molecule with an extremely short half-life; NO-releasing liposomes can prolong its duration of action and are effective against refractory non-tuberculous mycobacteria (*Mycobacterium abscessus*) biofilm infections. 3-methyl-1,2,3-oxadiazole-4-olate (MD3) is a small molecule NO-releasing prodrug capable of sustained NO release. Nagy et al. (2025) prepared liposomal MD3, which significantly improved biofilm inhibition and was able to eradicate biofilm bacteria at a concentration of 4 mg/mL.

Targeting *Acinetobacter baumannii*, Ji et al. (2023) demonstrated that the strongly positive charge residues of 2K4L promote electrostatic interactions with lipopolysaccharide (LPS) in the bacterial outer membrane, while its high hydrophobicity and α-helical conformation significantly increased membrane permeability of *A. baumannii*, providing a structural basis for antimicrobial peptide design. Shamkani et al. (2023) developed minocycline and gallium nitrate (GaN) co-loaded niosomes as biocompatible nanocarriers to enhance anti-biofilm properties against *A. baumannii* biofilms; niosomal encapsulation significantly enhanced the synergistic anti-biofilm efficacy of antibiotics and metal ions. Allemailem (2023) developed ellagic acid (EA) liposomal formulations to address the poor water solubility of ellagic acid, which exhibited anti-*A. baumannii* activity while simultaneously inhibiting biofilm formation, providing novel evidence for nanoformulations of natural polyphenolic compounds against biofilms. Khan et al. (2022) developed a whole-cell antigen liposome vaccine (Lip-WCAgs). Allemailem et al. (2021) investigated the *in vitro* antimicrobial and anti-biofilm activity of thymoquinone (TQ) against *A. baumannii*, prepared TQ liposomal formulations (Lip-TQ), and demonstrated that Lip-TQ possesses tremendous therapeutic potential with antimicrobial, antiinflammatory, and immunomodulatory properties.

Targeting *Klebsiella pneumoniae*, Akbarzadeh et al. (2023) employed computer-aided design combined with experimental validation to develop a gentamicin and EDTA coloaded liposomal drug delivery system, utilizing EDTA chelation of calcium and magnesium ions essential for maintaining biofilm integrity in synergy with gentamicin to kill drug-resistant *K. pneumoniae*.

Targeting *Listeria monocytogenes*, Zhang et al. (2025) developed a dual-loaded liposomal formulation of natural antimicrobial agents curcumin and nisin, combined with PDT, achieving a triple synergistic effect of chemical antibiosis, biofilm inhibition, and photoactivated bactericidal activity.

Targeting *Helicobacter pylori*, Sharaf et al. (2026) prepared a novel mucoadhesive nanocarrier COP-LIG@LIPSNCs, utilizing lignin to disrupt bacterial biofilms, enabling berberine liposomes to penetrate deeply and thereby exert enhanced therapeutic efficacy.

**Figure 7:**
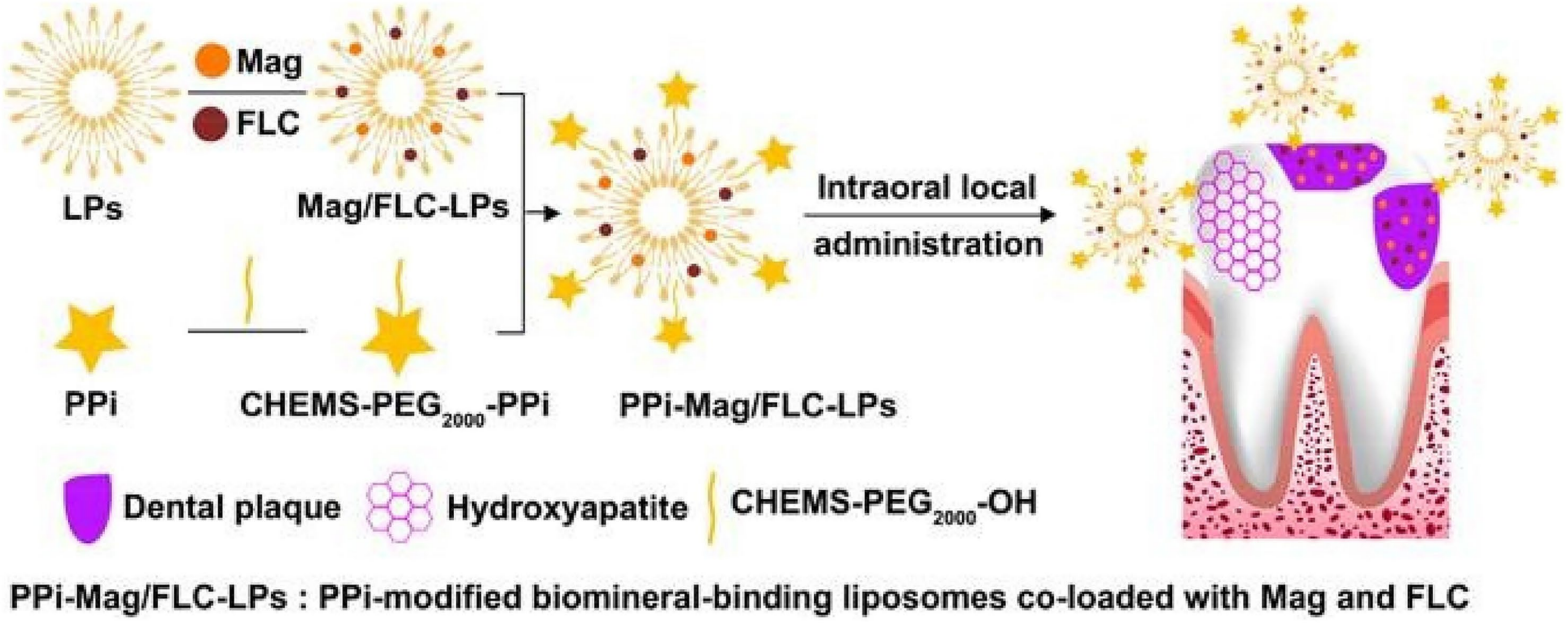
Mechanism diagram of biomineralization combined with liposomes loaded with Mag and FLC (Luo et al., 2023).

In the oral cavity, targeting *Streptococcus mutans*, Radmand et al. (2024) developed a nanoliposomal plant extract that effectively inhibits glucosyltransferase activity and disrupts bacterial virulence factors. Panda et al. (2024) developed a nano-liposomal formulation of modified photosensitizer toluidine blue O for anti-biofilm PDT. Targeting *Aggregatibacter actinomycetemcomitans*, Afrasiabi et al. (2023) developed doxycyclineloaded curcumin-doped liposomes (NL-Cur(+Dox)) for combined antimicrobial therapy against *A. actinomycetemcomitans*. Targeting *Porphyromonas gingivalis*, Han et al. (2024) developed chitosan-coated liposomes for delivery of antimicrobial peptide LL17-32. Targeting broad-spectrum oral bacteria such as dental plaque, Luo et al. (2023) constructed a liposomal drug delivery system PPi-Mag/FLC-LPs co-loading Mag and FLC, which not only achieved potent antimicrobial activity but also enhanced drug retention on tooth surfaces through binding to hydroxyapatite (Figure 7). Yang et al. (2023) prepared a novel cationic non-phospholipid nanoliposomal carrier, cetylpyridinium chloride/cholesterol CPC/Chol, exhibiting broad-spectrum antimicrobial activity. Xia et al. (2025) addressed the challenges of root canal treatment by developing a DNase I (for degrading biofilm eDNA) and PDT combined nano system, which overcomes *Enterococcus faecalis* biofilm barriers and addresses the problem of antibiotic penetration in pulp infections.

However, current oral biofilm research still heavily relies on *in vitro* models employing single-species or limited few-species configurations. Fernandez et al. noted that, constrained by limitations in early microbial identification techniques and driven by research interests targeting specific pathogenic behaviors, a substantial body of previous work has focused on single-species or small-consortium models. In reality, natural oral biofilms constitute dynamic ecosystems harboring hundreds of bacterial species, wherein the behavior of any individual species may be significantly altered by the presence of other community members, markedly diverging from its monoculture phenotype. Nithyanand et al. further emphasized that polymicrobial biofilms generate diverse virulence factors and exhibit more complex cross-resistance profiles, with clinical severity and antimicrobial resistance levels far exceeding those of single-species biofilms (Luo et al., 2022; Nithyanand et al., 2025).

### 3.2 Mechanisms of action

#### 3.2.1 EPS matrix penetration

The EPS matrix is the primary barrier in biofilms that impedes antibiotic penetration, with pore sizes of approximately 25–100 nm and carrying a negative charge. Liposomal nano-drug delivery systems enhance drug penetration capability primarily through the following mechanisms:

1. Size-dependent effect: Panthi et al. (2024) reviewed that liposomal nanoparticles with particle sizes below 100 nm significantly improved antibiotic delivery efficiency within biofilms, as smaller liposomes more readily traverse the EPS mesh structure. However, excessively small particle sizes (<30 nm) may lead to reduced drug encapsulation efficiency and accelerated systemic clearance; therefore, the optimal particle size range for anti-biofilm liposomes is considered to be 50–80 nm.
2. Surface charge regulation: Bacterial surfaces typically carry a negative charge; cationic liposomes utilize electrostatic attraction to enhance binding to bacteria. Nevertheless, cationic liposomes are susceptible to neutralization by serum proteins and may induce cytotoxicity. Makhlouf et al. (2023) reviewed the efficacy of various liposomal formulations against biofilms of Gram-negative and Grampositive bacteria, finding that PEG-modified liposomes prolong circulation time, while cationic modification enhances biofilm penetration. The optimal strategy involves balancing charge and stability based on the infection site and route of administration.
3. Enzyme-assisted penetration. Multiple studies have encapsulated EPS-degrading enzymes (such as DNase and dispersin B) within liposomes to disrupt matrix structure through enzymatic hydrolysis. Wang L. et al. (2024) employed a universal DBCO-azide bioorthogonal reaction to successfully synthesize novel phageliposome nanoconjugates. Utilizing phage components expressing depolymerases, the phage first promotes aggregation of the formulation on bacterial surfaces, then facilitates antibiotic entry into cells by disrupting bacterial cell walls. As expected, phage Sb-1 was also able to degrade the EPS of bioluminescent MRSA biofilms.

#### 3.2.2 Controlled release and targeting

The intelligent release mechanism of liposomes enables biofilm microenvironmenttriggered drug release, increasing local drug concentrations while reducing systemic exposure.

Due to bacterial acid metabolism, the interior of biofilms presents a lower pH value (5.56.5). Researchers have utilized this characteristic to develop a series of pH-sensitive targeted response systems. Wang G. et al. (2025) developed ultrasound-activated liposomes (LPCOTML) containing ROS-responsive lipid prodrugs (oleoyl-meropenem), which trigger release under acidic conditions of the biofilm microenvironment upon ultrasound irradiation. This system undergoes nanoparticle-microbubble phase transition under ultrasonic cavitation, mechanically disrupting the EPS structure to achieve deep penetration. Wang D. Y. et al. (2022) developed proton-responsive dual-drug (dispersant plus antibiotic) liposomes that trigger burst release in acidic biofilm microenvironments, first dispersing the biofilm then killing bacteria, thereby avoiding the risk of sepsis caused by high concentrations of dispersed bacteria. Deiss-Yehiely et al. (2023) developed pH-responsive charge-reversing nanoparticle surfaces that achieve charge reversal from positive to negative in acidic biofilm microenvironments, enhancing nanoparticle penetration within biofilms and drug delivery efficiency, overcoming the problem of excessive binding between traditional positively charged nanoparticles and biofilms.

Highly expressed enzymes within biofilms (such as hyaluronidase and phospholipase) can serve as trigger signals to achieve specific release of delivered drugs. Peng et al. (2024) utilized neutrophil-bacteria hybrid cell membrane vesicles to encapsulate lipid nanoparticles (LNP@HMVs), whose membrane surface enzymes can recognize and respond to the biofilm microenvironment. Mohammed et al. (2023) developed bacterial lipase-responsive vancomycin solid lipid nanoparticles (VCM-AS-SLNs) that exhibited excellent affinity for bacterial lipase; lipase significantly accelerated the release of loaded vancomycin, achieving targeted antibiotic delivery. Dong et al. (2024) developed lysozymeresponsive chitosan-coated liposomes (Lef@Lip@CS); *in vitro* antimicrobial experiments demonstrated significant efficacy differences before and after enzyme addition, with a 72.46% inhibition rate at 6 h, achieving on-demand drug release at infection sites. Ismail et al. (2024) developed dual-functional biomimetic liposomes (HAP3-Lipo) loaded with vancomycin free base (VCM) based on a novel TLR4-targeting peptide (P3) and hyaluronic acid (HA) for enhanced sepsis treatment, achieving a “targeting plus immune activation” synergistic effect.

Targeting the biofilm microenvironment, Xiao et al. (2025) designed a liposomal drug delivery nanoparticle (ICG-rapamycin) encapsulating indocyanine green (ICG) and rapamycin. ICG-rapamycin elevates ROS levels and temperature under near-infrared (NIR) laser irradiation, promoting PDT and photothermal therapy (PTT) antimicrobial mechanisms. Simultaneously, it prevents biofilm formation by increasing ATP levels. Through immune regulation via three aspects—promoting macrophage M2 polarization, upregulating anti-inflammatory factor TGF-*β*, and enhancing macrophage phagocytosis of bacteria—it achieves long-term inhibitory effects on biofilm formation. Chen et al. (2023a) developed a nano-drug delivery system Lipo/Van@Arg loaded with vancomycin and surface-linked with positively charged L-arginine. Lipo/Van@Arg exhibited excellent bacterial binding and biofilm penetration capabilities; the unique H₂O₂-triggered nitric oxide release mechanism of this formulation enhanced bactericidal efficacy. Loscertales and España (2026) optimized liposomal membrane composition by regulating the ratio of polyunsaturated fatty acid phospholipids to saturated fatty acid phospholipids, enhancing radiosensitivity and colloidal stability. Meanwhile, they introduced a radiosensitization mechanism based on Fe^3+^ ion encapsulation, where Fe^3+^ ions are reduced to Fe^2+^ upon γray irradiation. This redox transformation triggers a Fenton-like reaction, catalyzing the degradation of lipid H₂O₂, leading to local lipid peroxidation and membrane disruption. This dual strategy of membrane composition regulation combined with iron-mediated oxidative activation significantly enhanced drug release under low-dose radiation exposure. Yang et al. (2024) developed redox liposomal nanobombs by simultaneously introducing heme with peroxidase-like catalytic activity and its substrate ABTS into liposomes. High H₂O₂ levels at infection sites or within biofilm microenvironments trigger a cascade reaction, generating abundant alkyl radicals that efficiently eradicate biofilm bacteria.

Thermal-responsive and photothermal-responsive strategies represent a hot research area. Zhao et al. (2023) developed thermosensitive liposomal nanoparticles (TLCA) with positive charge and particle sizes below 230 nm that can effectively diffuse into biofilms. Combined photothermal ablation and chemotherapy effectively addressed the therapeutic challenge of bacterial biofilm formation on necrotic bone surfaces in MRSA-infected osteomyelitis. Xu et al. (2020) constructed a near-infrared controllable antimicrobial nanoplatform based on tungsten disulfide quantum dots (WS₂QDs) and vancomycin loaded in thermosensitive liposomes. This system utilizes the photothermal sensitivity of WS₂QDs to achieve selective rupture of liposomes, thereby enabling targeted drug delivery; the elevated temperature also enables WS₂QDs to exert multiple enzymemimicking activities, thereby enhancing antimicrobial efficacy. Jiang et al. (2025) combined photosensitizer CyI and small-size platinum nanoparticles (Pt NPs) into thermosensitive liposomes, and modified maltooligosaccharides on the liposome surface, preparing a bacterial targeting liposomal nanoplatform MCPL. MCPL specifically targets bacterial cell membranes; after thermal activation, it catalyzes the conversion of endogenous H₂O₂ to O₂, providing an additional substrate pool for PDT to generate ROS, thereby achieving stronger antimicrobial effects. Furthermore, MCPL can regulate HIF1α and NF-κB signaling pathways in immune cells. Chen et al. (2023b) developed copper oxide nanoparticle-loaded liposomes Ce6@Lipo/UCONs; their cationic properties enable penetration of MRSA biofilms, and responsive release of loaded UCONs in the acidic biofilm microenvironment produces PDT killing and antimicrobial effects.

Due to the fact that photodynamic therapy (PDT) and photothermal therapy (PTT) do not induce bacterial resistance, these modalities have garnered considerable attention from researchers and clinicians in recent years. Building upon the aforementioned studies, scholars have identified that traditional photosensitizers (e.g., porphyrins and phthalocyanines) frequently suffer from aggregation-caused quenching (ACQ) in dense biological environments, resulting in diminished fluorescence signals and reduced reactive oxygen species (ROS) production. In contrast, aggregation-induced emission (AIE) photosensitizers exhibit enhanced fluorescence emission and ROS generation upon aggregation, a characteristic that renders them particularly advantageous in biofilm microenvironments rich in extracellular polymeric substances (EPS). Wu et al. (2024) systematically reviewed targeted antibacterial photodynamic therapy strategies based on AIE photosensitizers and proposed the concept of “sheltered bacteria,” which encompasses biofilm-embedded bacteria, intracellular bacteria, and abscess-residing bacteria. These sheltered bacteria evade the killing effects of conventional therapeutic modalities by virtue of the EPS barrier effect. The authors further elaborated on strategies including cationic-hydrophobic dual effects, ligand/phage-mediated targeting, chemically responsive activation, metabolic labeling, and multifunctional adoptive cell transfer, providing a comprehensive description of precision theranostic regimens under the paradigm of integrated diagnosis and treatment (Wu et al., 2024).

Furthermore, Wang G. et al. (2025) designed an ultrasound-activated liposome with transcytosis capability that can cross epithelial barriers; ultrasound-triggered drug release combined with deep penetration of a dense EPS matrix effectively addressed the problem of insufficient drug accumulation and penetration in biofilm-associated surgical site infections (BSSI).

Ashar et al. (2023) combined the aforementioned targeting and controlled-release strategies, developing a “thermal targeting, on-demand” antibiotic delivery method by combining ciprofloxacin-loaded thermosensitive liposomes with focused ultrasound, to address the problem of reduced antibiotic sensitivity in chronic bone infections caused by *S. aureus* biofilms in children and adults, achieving precise controlled-release bactericidal action. Furthermore, Mahafel et al. (2024) developed 4-nitroimidazole-modified porphysomes loaded with pistachio green hull extract (PGHE) by combining pH and temperature responsive release, providing a new strategy for natural product-synthetic drug synergistic antimicrobial therapy.

Gentili et al. (2023) compared the antimicrobial efficacy of liposomes containing ozonated sunflower oil with commonly used ophthalmic disinfectants (povidone-iodine, chlorhexidine). Liposome-encapsulated ozonated oil demonstrated efficacy in reducing biofilm formation, decreasing selection pressure for antibiotic-resistant bacteria, and reducing bacterial adhesion to corneal cells. Wang et al. (2021) prepared cinnamaldehydeloaded liposomes modified with different concentrations of chitosan and evaluated their physicochemical properties and antimicrobial performance. Results showed that physical modification with chitosan improved liposome encapsulation efficiency and storage stability, and exerted cumulative and synergistic bacteriostatic effects, leading to damaged cell membrane integrity and induced cell death through intracellular component leakage. Hu et al. (2025) developed a tetrahedral framework nucleic acid (tFNA)-based antibiotic nano-drug delivery system that effectively controlled bacterial load and alleviated inflammatory responses in biofilm-associated skin and lung infection mouse models without visible toxicity. Li et al. (2026) developed a biphasic liposome platform lip@Lys/NC that produces bactericidal and bacteriostatic effects from three aspects: bacterial lysis, quorum sensing inhibition, and anti-inflammation. Motavaf et al. (2025) prepared selenium nanoparticle (SeNP) liposomal formulations that exhibited stronger antimicrobial and anti-biofilm activity compared to free SeNPs. Lee et al. (2026) prepared nanostructured NLCs loaded with minocycline, with particle sizes reaching 81 and 76 nm, effectively inhibiting the viability of Cutibacterium acnes in both planktonic and biofilm forms, providing a new delivery strategy for transcutaneous acne treatment.

Through nanonization of mucolytic agents, Pinto et al. (2021) developed N-acetyl-Lcysteine (NAC)-loaded nanosystems to address the health problem of increased antibiotic resistance caused by bacterial biofilms. *In vitro* anti-biofilm efficacy testing demonstrated effectiveness against both *Staphylococcus epidermidis* (Gram-positive) and.

*Pseudomonas aeruginosa* (Gram-negative). In another study, Wang J. et al. (2024) developed a muco-inert ciprofloxacin-colistin co-loaded liposome (Cipro-Col-Lips) dry powder inhalation formulation that effectively penetrates airway mucus and accumulates at biofilm sites, neutralizing toxins and protecting lung cells. Triggered release of ciprofloxacin and colistin synergistically reduces biofilm antibiotic resistance.

Modification of liposome components with natural products can also effectively enhance bacteriostatic efficacy. Bharathi et al. (2024) prepared linoleic acid (LA)-loaded liposomes that effectively disrupted pre-formed biofilms of *Staphylococcus aureus* and *Candida albicans*. The formulated liposomal LA (0.1 g/mL) exhibited 100-fold enhanced dual biofilm inhibitory activity compared to LA alone, providing data on the dose-effect relationship of fatty acid liposomes against biofilms. Deol et al. (2024) developed sesamolloaded solid lipid nanoparticles (SLNs) that promote wound healing by inhibiting bacterial growth, eliminating bacterial biofilms at wound sites, and regulating oxidative stress in skin tissue, providing new evidence for nanonization of natural phenolic compounds. Sun et al. (2025) combined microalgae *Chlorella pyrenoidosa* with antibiotic liposomal gel, achieving a dual wound healing strategy of “clearance plus repair” by inhibiting biofilm formation through gene regulation while promoting tissue regeneration.

### 3.3 Synergistic antimicrobial effects

Liposomes can simultaneously encapsulate multiple antimicrobial agents to enhance efficacy through synergistic mechanisms:

Antibiotic-antimicrobial peptide combinations. Alzahrani et al. (2022) co-encapsulated tobramycin and IDR-1018 anti-biofilm peptides within liposomes, significantly reducing *P. aeruginosa* biofilm formation at concentrations ≥4 μg/mL. The IDR-1018 peptide enhances tobramycin penetration by disrupting bacterial membrane integrity; the synergistic interaction between the two drugs increased biofilm clearance by more than three-fold. Shao et al. (2023) constructed cationic antimicrobial peptide (AMP)-conjugated liposomes with virus-like structures. Targeting the prevalence of multidrug-resistant pathogens and the emergence of biofilms, this system utilizes the unique non-specific membrane disruption mechanism of AMPs to enhance antimicrobial efficacy and biosafety.

Antibiotic-phage combinations. Wang L. et al. (2024) developed phage-liposome nanoconjugates that combine the specific lytic capability of phages with the drug delivery advantages of liposomes (Renner and Weibel, 2011). In a rat prosthetic joint infection model, this system effectively reduced bacterial load and promoted osteomyelitis recovery, providing a new strategy for orthopedic implant-associated infections.

Antibiotic-natural compound combinations. Alluhaim et al. (2024) utilized Manuka honey as a surfactant to develop methylglyoxal-tobramycin liposomes (Lip-MGO-TOB), reducing *P. aeruginosa* biofilm formation by 68% at 32 μg/mL (compared to only 21% for free tobramycin). Methylglyoxal possesses broad-spectrum antimicrobial activity and synergizes with tobramycin to enhance therapeutic efficacy. Dias-Souza et al. (2025) coencapsulated rhamnolipids and antibiotics in liposomes for controlling planktonic and biofilm-associated bacteria with low antimicrobial sensitivity in cooling towers, demonstrating that liposomal encapsulation significantly enhanced antimicrobial efficacy. Gelen-Gungor et al. (2025) developed azithromycin-nisin co-delivery liposomes. Evaluation of antimicrobial activity and biofilm clearance against *S. aureus* demonstrated that combined delivery significantly reduced antimicrobial drug resistance. Alzahrani et al. (2024) developed gentamicin-thymoquinone co-encapsulated liposomes (Lipo-GEN-THQ). Transmission electron microscopy was employed to evaluate bacteria-liposome interactions. Liposomes exhibited good stability and sustained gentamicin release over 24 h, with antimicrobial activity significantly superior to free gentamicin.

Antibiotic-adjuvant combinations. Rao et al. (2022) designed a hypoxia-sensitive antibiotic-adjuvant liposome (NANO@PS-LPs), co-encapsulating azithromycin (AZI), adjuvant (2-nitroimidazole derivative, 6-NIH), and biofilm dispersant (nitric oxide donor, DETA NONOate). NANO@PS-LPs possess a negatively charged surface and good hydrophilicity, enabling easy penetration through the sputum layer. Subsequently, phospholipase A2 (PLA2) overexpressed in the microenvironment around biofilms triggers its disassembly. Nitric oxide produced by DETA NONOate promotes dispersion of *P. aeruginosa* biofilms, while 6-NIH is reduced to 2-aminoimidazole derivative (6AIH) under hypoxic conditions, functioning as an AZI adjuvant to enhance antimicrobial activity. This system represents a promising nanostrategy for treating refractory *P. aeruginosa* pulmonary infections and overcoming associated resistance. Alarfaj et al. (2022) developed tobramycin-N-acetylcysteine (TNL) liposomal formulations targeting tobramycin-resistant bacterial strains. Assessment of antimicrobial activity and biofilm reduction demonstrated that compared to tobramycin (TL) liposomes, TNL enhanced antibiotic penetration. Gerayelou et al. (2021) developed nanoliposomal formulations for co-delivery of vancomycin and cis-2-decenoic acid (C2DA) to *S. epidermidis* biofilms. The combination of vancomycin and C2DA inhibited biofilm formation, demonstrating that liposomal composite formulations are a viable approach for bacterial biofilm clearance.

Protease-natural product combinations. Tsai et al. (2023) developed multifunctional cationic liposomes co-loaded with proteinase K (PK) and retinoic acid (SME), exhibiting synergistic inhibitory effects against Cutibacterium acnes biofilm colonization, and reducing adherent bacterial colonies in microplates. This system provides an enzymedrug combination nanoplatform for acne treatment.

Natural product combinations. Pashizeh et al. (2024) developed gingerol-loaded alginatecoated niosomes (Gin-Nio@AL), whose biofilm formation inhibitory effect was significantly superior to Gin-Nio and free gingerol, providing a new strategy for natural product-polysaccharide synergistic anti-bacterial nanoplatforms. Pu et al. (2026) demonstrated that natural terpenoid compounds (geraniol and citral) exhibit synergistic activity when co-encapsulated. The two compounds were incorporated into liposomes (G/C liposomes) using the ethanol injection method, overcoming their poor water solubility and stability while enhancing antimicrobial efficacy. The therapeutic potential of this system has been validated both *in vitro* and *in vivo*. Luo et al. (2021) reported a multiple surface modification strategy—utilizing PEG to prolong circulation, tea saponin to enhance membrane interactions, and dihydromyricetin to inhibit bacterial energy metabolism—targeting the oxidative respiratory chain of bacteria within biofilms, achieving a dual mechanism of “metabolic inhibition plus physical destruction.”

### 3.4 Photothermal combination therapy

Zhu et al. (2026) utilized microfluidic technology to prepare an Mg^2+^-chelated microgel GD Lip@Mg. The main component of this microgel is liposomes loaded with DF-Cur and glycyrrhizic acid (GA). GA inhibits the bacterial stress response chaperone protein HSP60, thereby sensitizing MRSA to DF-Cur-mediated PTT. Under 450 nm laser excitation, bacterial numbers were reduced by >99.9%, while simultaneously increasing re-epithelialization, collagen deposition, and vascular density, and shifting macrophages from M1 to M2 phenotype without thermal damage. Results demonstrated effective eradication of *methicillin-resistant Staphylococcus aureus* (MRSA) and promotion of wound healing. Song et al. (2025) developed Au/PDA/HRP@DLP multifunctional nanocatalysts by encapsulating horseradish peroxidase (HRP)-loaded polydopaminemodified gold nanoparticles in DOTAP/DOPE cationic liposomes, achieving photothermal-sonodynamic synergistic antimicrobial action and immune reprogramming for effective treatment of chronic osteomyelitis. Cressey et al. (2022) developed phospholipid-porphyrin conjugate liposomes with dual photothermal and photodynamic activity, exhibiting nearly identical PTT effects against both planktonic and biofilmassociated *S. aureus* and *P. aeruginosa*. Yan et al. (2023) rationally designed three butterfly-shaped aggregation-induced emission molecules (AIEgens) that balance nonradiative decay (for PTT) and radiative decay (for NIR-II optical window fluorescence imaging). Encapsulated in cationic liposomes, they were used for imaging-guided photothermal ablation of bacterial biofilms.

### 3.5 Engineered formulation combination therapy

Tang et al. (2025) combined *Bdellovibrio bacteriovorus* with nanotechnology. Engineered *B. bacteriovorus* can prey on Gram-negative bacteria and penetrate biofilms, thereby enhancing antibiotic penetration efficiency. Pourtalebi Jahromi et al. (2026) developed hybrids of bacterial-mimetic liposomes with biocompatible myxobacterial outer membrane vesicles (OMVs) as a controllable platform for targeted antibiotic delivery, providing a new strategy for treating intestinal biofilm infections. Drost et al. (2021) utilized a bacterial biomimetic strategy to prepare liposomes composed of DOPG (18:1 (Δ9-cis) phosphatidylglycerol) and CL (cardiolipin), mimicking the cell membranes of Grampositive bacteria *S. aureus* and *Streptococcus pneumoniae*, and loaded a novel hydrophobic compound to effectively produce antimicrobial effects.

Notably, the fusogenic liposomes discussed within the aforementioned targeted modification strategies have been engineered to transition from conventional drug carriers to membrane fusion-mediated intracellular delivery systems. By mimicking viral membrane fusion mechanisms, these liposomes can directly fuse with bacterial cell membranes, thereby delivering encapsulated antibiotics (e.g., vancomycin) into the bacterial cytoplasm and significantly enhancing the antimicrobial efficacy against MRSA and other drug-resistant bacteria (Okafor et al., 2025). Studies by Scriboni et al. (2019) have confirmed that fusogenic vancomycin-loaded liposomes exhibit significantly superior bactericidal activity against mature MRSA biofilms compared to conventional liposomes, while Tat peptide-functionalized and pH-responsive fusogenic liposomes further expand the applicability of this strategy to Gram-negative bacteria and meningeal infections. This structure–function optimization strategy offers an important direction for further research on pathogen-specific liposomes; nevertheless, the precise regulation of *in vivo* safety and fusion efficiency remains a critical challenge for future studies (Nithyanand et al., 2025).

### 3.6 Biomineralized nanocarriers and lipid–inorganic hybrid delivery systems

Although liposomes have achieved remarkable clinical success as classical biomimetic drug delivery platforms (e.g., with the approval of pioneering nanomedicines such as Doxil^®^), their broader application remains constrained by inherent physicochemical limitations, including insufficient storage stability, drug leakage tendencies, and inadequate resistance to environmental stresses. In recent years, biomineralized nanocarriers inspired by natural biomineralization processes have emerged as a highly promising complementary strategy to address these bottlenecks (Dong et al., 2023).

Schmidt et al. (2004) utilized anionic phospholipid (DOPA) liposomes as templates to achieve controlled growth of calcium phosphate mineralization layers on the liposome surface through electrostatically induced Ca^2+^ deposition. Xu et al. (2007) further developed hydroxyapatite (HA)-mineralized liposomes (HACL), enabling extended drug release periods and pH-responsive release characteristics tailored to the acidic tumor microenvironment.

Furthermore, biomineralization strategies have recently been extended to more sophisticated hybrid system designs. Li et al. (2010) developed lipid-coated biodegradable calcium phosphate nanoparticles, successfully achieving systemic siRNA delivery; Wu et al. (2017) designed novel lipid-coated calcium phosphate–carbonate hybrid nanoparticles that exploit endosomal acidic environments to trigger drug release, significantly enhancing gene delivery efficiency. Additionally, Wang Y. et al. (2022) integrated calcium carbonate–lipid dual membranes with mesoporous silica to construct a delayed-release system, enabling refined modulation of drug release kinetics through the synergistic effect of multilayer mineralization barriers.

On the other hand, biomineralized nanocarriers possess inherent stimuli-responsive degradation properties. For instance, calcium carbonate nanoparticles remain stable under neutral pH conditions while decomposing in acidic environments to generate CO₂ bubbles, thereby serving dual functions of drug release and ultrasound imaging contrast enhancement.

Recently, Wang et al. reported a tea polyphenol-based biocompatible nanoplatform that achieves efficient cytosolic delivery of protein therapeutics through multiple noncovalent interactions between natural polyphenols and proteins, significantly reducing the immunogenicity and cytotoxicity of the carrier itself while maintaining protein activity. By the same token, their proposed engineering concept of constructing highly biocompatible carriers from natural polyphenol-based materials and promoting endosomal escape through membrane interactions can be directly translated to the construction of next-generation liposomal antibacterial platforms. For example, embedding or modifying tea polyphenols or similar natural polyphenols on liposome surfaces is expected to prolong their circulation half-life, enhance the transmembrane delivery efficiency of antibacterial agents, and enable the construction of multifunctional composite carriers when integrated with existing strategies. Furthermore, by the same rationale, fluorinated polymers can be incorporated. Leveraging their unique fluoroamphiphilic effect, these polymers enable efficient direct membrane translocation and cytosolic release without compromising cell membrane integrity, exhibiting significantly superior tissue penetration depth and intracellular delivery efficiency compared to conventional alkylated carriers. Boronated polymers, in turn, achieve deep binding with protein surfaces through N–B coordination and guanidinium–*π* interactions, and can trigger reversible dissociation in acidic microenvironments, providing a chemical switch for the precise release of antibacterial agents. Guanidinium-functionalized polymers, meanwhile, significantly enhance the membrane translocation activity and intracellular uptake efficiency of nanocarriers through stable salt bridges formed between strongly basic guanidinium groups and cell membrane phospholipids as well as protein carboxyl groups. The incorporation of these functional ligands into liposome surface modification is expected to overcome the limitations of conventional liposomes in intracellular delivery efficiency and targeting specificity. It is worth emphasizing that the clinical translation of the aforementioned engineering strategies still requires addressing critical issues such as the reproducibility of large-scale production, long-term *in vivo* safety monitoring, and standardized characterization of carrier–drug interactions. With the deep interdisciplinary integration of materials science, chemistry, and infectious disease research, next-generation liposomal nanoplatforms that combine high biocompatibility, efficient intracellular delivery, and intelligent responsiveness will offer more clinically translatable therapeutic strategies for refractory biofilm-associated infections and intracellular bacterial infections (Wang H. et al., 2025).

### 3.7 Other strategies

Tort et al. (2024) prepared proliposomes, embedding liposome precursors in nanofibers that *in situ* hydrate to form liposomes upon contact with body fluids. This method addresses the limitations of long-term stability of conventional liposomes, while retinoic acid confers antimicrobial and pro-repair functions. Almeida Campos et al. (2024) utilized liposomes to encapsulate plant lectin CrataBL, reducing its cytotoxicity while preserving antistaphylococcal, anti-biofilm, and anti-Trypanosoma cruzi activities.

Bidaki et al. (2025) utilized 3D printing technology to construct a multilayer composite scaffold—combining chitosan with alginate and a traditional Iranian herb Barijeh loaded in liposomes—to create Nio-Bar@CS-AL. This system achieved >99.999% bactericidal efficacy and significant biofilm inhibition, exhibiting potent antimicrobial activity.

Bile salt-modified liposome strategies. Bile salts as natural permeation enhancers address the problems of poor oral absorption and short half-life of hydrophilic cephalosporin antibiotics. A bile salt sodium deoxycholate modification can enhance membrane fluidity; when combined with carboxymethyl cellulose hydrogel, this system overcomes the physical barriers posed by biofilms (Rashid et al., 2025; Abdullah et al., 2025).

### 3.8 Quorum sensing interference

Quorum sensing (QS) is a communication mechanism through which bacteria coordinate collective behavior via signaling molecules, regulating biofilm formation and virulence factor expression. Liposomes can deliver quorum sensing inhibitors to interfere with bacterial communication (Hamoud et al., 2025; Nyffeler et al., 2022; Dolph et al., 2025). Sources of quorum sensing blockers include AHL analogues, furanone derivatives, and enzymatic degradation strategies. Nosair et al. (2025) discovered that the virulence factor staphyloxanthin (STX) of *S. aureus* could be repurposed through vesicle encapsulation, enabling it to inhibit quorum sensing, biofilm formation, and persister cells of *Acinetobacter baumannii*—a novel application of fighting poison with poison.

### 3.9 Clinical studies and approved innovative drugs

Multiple liposomal antibiotic formulations are currently in clinical use or under investigation; these are summarized in Table 1.

**Table 1:** Summary of liposomal antibiotic formulations for bacterial infections.

Drug name	Drug class	Indication/Target pathogen	Clinical status	Key features/Advan tages	References
Amikacin Liposome Inhalation Suspension (ALIS)	Aminoglyco side (Amikacin)	Non-tuberculous mycobacteri a (NTM) pulmonary infection, resistant strains	Approved (USA, etc.)	Targeted pulmonary delivery, reduced systemic toxicity	Griffith et al. (2018)
Amphotericin B Liposome	Polyene (Amphoteric in B)	Fungal infections (some exploration for bacterial infections)	Approved	Reduced nephrotoxicity, enhanced efficacy	Walsh et al. (n.d.)
Colistin Liposome	Polypeptide (Colistin)	Multidrugresistant Gram-negative bacteria (e.g., *A. baumannii*, etc.)	Preclinical/Investig ational	Enhanced pulmonary concentration, reduced nephrotoxicity	Karpuz et al. (2023)
Ciprofloxacin Liposome	Fluoroquino lone (Ciprofloxac in)	Refractory respiratory bacterial infections	Clinical trial	Prolonged drug action, targeted delivery	Bassetti et al. (2020); Cipolla et al. (2016)
Moxifloxacin Liposome	Fluoroquino lone (Moxifloxac in)	*S. aureus*, *S. epidermidis*, etc.	Preclinical/Early clinical	Enhanced antimicrobial activity, improved pharmacokinet ics	Natsaridis et al. (2025)
Vancomycin Liposome	Glycopeptid e (Vancomyci n)	*MRSA* and other resistant Gram-positive bacteria	Preclinical/Early clinical	Enhanced tissue penetration, reduced nephrotoxicity	Erdene et al. (2025)
Azithromycin Liposome	Macrolide (Azithromyc in)	*Chlamydia trachomatis*, etc.	Preclinical/Early clinical	Enhanced intracellular delivery, improved efficacy	Yıldırım and Düzgüneş (2025)
Berberine liposome	Plant alkaloid (Berberine)	Resistant gram-negative bacteria	Preclinical	Enhanced bioavailability, auxiliary anti-resistance activity	Costa et al. (2024)

ALIS represents the most successful clinical translation of a liposomal anti-biofilm strategy to date, approved by the FDA in 2018 for refractory *Mycobacterium avium* complex (MAC) lung disease. Its success hinges on two strategic choices. First, the inhalation route bypasses systemic exposure, predominantly enriching drug concentrations in pulmonary airways and macrophages, thereby avoiding the nephrotoxicity and ototoxicity associated with aminoglycosides. Second, liposomal nanodrug delivery systems (NDDS) enable macrophage-targeted delivery and enhanced penetration into non-tuberculous mycobacterial (NTM) biofilms, advantages that free amikacin does not possess.

Liposomal colistin remains in clinical trials or regionally approved status. Clinical trials have shown that conventional colistin is associated with high rates of early acute kidney injury (AKI), particularly in critically ill patients (Duszynska, 2025), while liposomal nanodrug delivery systems (NDDS) have demonstrated the potential to reduce nephrocytotoxicity in preclinical models by shielding renal tubular cells from direct exposure. However, the advancement of liposomal colistin programs has been slowed by the need for higher drug loading to achieve sufficient colistin release within dense biofilms, coupled with formulation challenges arising from the difficulty of effectively encapsulating polymyxins into conventional liposomes (Wang et al., 2018).

Formulations such as moxifloxacin, azithromycin, and berberine liposomes remain in preclinical or early clinical stages, hindered by a common set of translational barriers.

Foremost is the lack of standardized, clinically predictive biofilm infection models. Current animal models often fail to replicate the complexity of human biofilm infections, including multispecies composition, host immune responses, and anatomical fluid dynamics.

In summary, the current clinical challenges are as follows:

1. Disconnect between microbiological and clinical efficacy: Culture conversion does not directly correlate with symptomatic relief or recurrence, as certain variant or residual strains may remain undetected.
2. Regulatory complexity: Liposomal antibiotics are classified as complex drug products; achieving robust batch-to-batch consistency and other quality standards results in extended development timelines and substantial costs.
3. Long-term safety monitoring: The safety profile of chronic administration remains to be fully evaluated.

## 4 Summary and outlook

Chronic infections caused by bacterial biofilms and antimicrobial drug resistance have become major global public health challenges, with biofilm formation involved in over 65% of bacterial infections (Jamal et al., 2015). Traditional antibiotic therapy faces three critical bottlenecks: physical barrier of the EPS matrix, persister cell formation due to bacterial metabolic heterogeneity, and horizontal transfer of resistance genes.

With deepening research, emerging liposomal delivery systems have demonstrated fundamental distinctions from conventional liposomes in recent years:

1. Membrane fusion and intracellular delivery. Classical liposomes rely on endocytosis for cellular entry, resulting in extensive drug sequestration within endo-lysosomal compartments and subsequent inactivation. This severely compromises their capacity to target intracellular persisters. Engineered fusogenic liposomes, by contrast, completely bypass endocytosis and directly fuse with bacterial membranes to deliver drugs into the cytoplasm.
2. Drug loading and storage stability. Traditional liposomes are more susceptible to poor storage stability and drug leakage. Recently developed biomineralized hybrids and proliposome formulations circumvent these limitations by introducing inorganic mineral barriers or enabling in-situ hydration of dehydrated precursors, transforming unstable single-compartment vesicles into robust multi-barrier delivery systems.
3. Targeting efficiency and biofilm penetration. Unmodified conventional liposomes frequently adhere excessively to biofilm surfaces, obstructing deep penetration. Intelligent designs, such as pH-responsive charge-reversing nanoparticles and hybrid membrane vesicles, can dynamically adapt to biofilm microenvironments.
4. Therapeutic efficacy and functional integration. Conventional nanodrug delivery systems (NDDS) typically deliver single antibiotics, whereas modern platforms integrate multi-modal synergistic strategies by co-encapsulating antibiotics with adjuvants, photothermal/photodynamic modules, or quorum sensing inhibitors. This approach resolves classical bottlenecks related to penetration depth, metabolic heterogeneity, and persister cell survival.

Future technological development is mainly manifested in three aspects. 1. Precision targeting: Current antibody modification methods are costly and potentially immunogenic; future efforts should focus on developing novel ligands, such as bacteria-specific aptamers and phage tail fiber proteins, combined with AI-assisted screening, to achieve “one bacterium, one strategy” precision therapy (Chen et al., 2026). 2. Multi-stimuli responsiveness: Single-stimuli responsive systems have inherent limitations; systems such as pH-enzymeultrasound triple-responsive systems can be constructed to achieve spatiotemporal cascade control (Farah et al., 2026). 3. Theranostics: Loading near-infrared fluorescent dyes or magnetic nanoparticles enables real-time monitoring of biofilm dispersal, guiding individualized adjustment of treatment duration to avoid overtreatment or undertreatment (Chen et al., 2026).

Clinical translation is another major challenge following basic research. The challenges faced in current clinical translation and relatively feasible solution strategies are summarized in the Table 2.

**Table 2:** Challenges in clinical translation and solutions.

Current bottleneck	Solution strategy
Batch quality control for scale-up production	Adopt microfluidic continuous production technology; establish a quality standard system (Maeki et al., 2022)
Insufficient storage stability	Optimize lyoprotectant formulation; develop ready-to-use prefilled formulations emphasizing that rigorous stability monitoring—including particle size, polydispersity index, zeta potential, and drug retention as well as strategies such as lyophilization with cryoprotectants (e.g., trehalose or sucrose), metal ion chelation, antioxidant incorporation, and stringent temperature-controlled storage (Geng et al., 2026)
Rapid clearance by the mononuclear phagocyte system (MPS)	Explore “low-PEG” alternatives (e.g., CD47 “do not eat me” signal modification) (Xie et al., 2025)
Current Bottleneck	Solution Strategy
Scarce clinical data	Establish standardized biofilm infection animal models; conduct investigator-initiated clinical trials (IITs) (Farah et al., 2026)

Finally, with the development of science and technology, exploration can be conducted in emerging interdisciplinary fields, such as immunotherapy combination, resistance surveillance systems (Makhlouf et al., 2023), AI-driven design (Farah et al., 2026), etc.

Over the past five years, liposomal anti-biofilm research has advanced from proof-ofconcept to mechanism deepening and functional innovation. The development of intelligent responsive liposomes, biomimetic surface engineering, and composite carrier systems has provided powerful tools for overcoming the therapeutic dilemma of chronic infections. Nevertheless, the journey from laboratory to clinic remains lengthy. This will require collaborative efforts from materials scientists, microbiologists, clinicians, and regulatory agencies. Future research should focus on precision, intelligence, and immune synergy, driving liposome technology to become a true clinical weapon against biofilmassociated infections.
